# Rome Criteria and a Diagnostic Approach to Irritable Bowel Syndrome

**DOI:** 10.3390/jcm6110099

**Published:** 2017-10-26

**Authors:** Brian E. Lacy, Nihal K. Patel

**Affiliations:** Dartmouth-Hitchcock Medical Center, One Medical Center Drive, Lebanon, NH 03756, USA; brian.e.lacy@hitchcock.org

**Keywords:** Rome criteria, irritable bowel syndrome, IBS

## Abstract

Functional gastrointestinal disorders (FGIDs) account for at least 40% of all referrals to gastroenterologists. Of the 33 recognized adult FGIDs, irritable bowel syndrome (IBS) is the most prevalent, with a worldwide prevalence estimated at 12%. IBS is an important health care concern as it greatly affects patients’ quality of life and imposes a significant economic burden to the health care system. Cardinal symptoms of IBS include abdominal pain and altered bowel habits. The absence of abdominal pain makes the diagnosis of IBS untenable. The diagnosis of IBS can be made by performing a careful review of the patient’s symptoms, taking a thoughtful history (e.g., diet, medication, medical, surgical, and psychological history), evaluating the patient for the presence of warning signs (e.g., “red flags” of anemia, hematochezia, unintentional weight loss, or a family history of colorectal cancer or inflammatory bowel disease), performing a guided physical examination, and using the Rome IV criteria. The Rome criteria were developed by a panel of international experts in the field of functional gastrointestinal disorders. Although initially developed to guide researchers, these criteria have undergone several revisions with the intent of making them clinically useful and relevant. This monograph provides a brief overview on the development of the Rome criteria, discusses the utility of the Rome IV criteria, and reviews how the criteria can be applied clinically to diagnose IBS. In addition, a diagnostic strategy for the cost-effective diagnosis of IBS will be reviewed.

## 1. Case Study

L.J. is a 32 year-old woman referred for a second opinion in gastroenterology. Gastrointestinal symptoms began 5 years ago after a trip to Mexico. Both she and her husband suffered the acute onset of nausea, vomiting, abdominal pain and diarrhea after eating at a seaside resort. Several other dinner guests developed similar symptoms. It was thought that they had food poisoning, and conservative measures (clear liquids for 2 days, p.r.n. acetaminophen, p.r.n. antiemetics) were employed. Her husband’s symptoms slowly resolved and his bowel habits returned to normal. However, since that time, she has been troubled with recurrent episodes of lower abdominal pain combined with a sense of fecal urgency, tenesmus, and loose, watery, non-bloody stools. She frequently feels bloated and distended and states that she looks “4 months pregnant”. She saw her internist shortly after her return from Mexico. Stool studies (ova and parasites, fecal leukocytes, routine stool cultures) were performed and were normal. Blood work was also normal (complete blood count or CBC, complete metabolic panel or CMP, and C-reactive protein or CRP were normal). She was told that this was likely just an after effect of a prior infection and that symptoms would slowly resolve. However, when her symptoms did not resolve, she saw another internist. Serologic tests (serum TTG or tissue transglutaminase antibody and serum IgA) to look for celiac disease were negative. Separate one-month long trials of avoiding dairy, then fructose, and then gluten did not help. Her weight remained stable and no new symptoms developed. She was told that her symptoms would slowly resolve and that she should “just learn to eat around it”. Frustrated by her persistent symptoms, she sought out the advice of a gastroenterologist. A review of symptoms did not elicit any new meaningful information. Her physical examination was normal. Repeat blood work (CBC, CMP, and CRP) and a thyroid test (TSH) all returned normal. A colonoscopy was performed and was grossly normal; random biopsies of the terminal ileum and colon were all normal. The patient was told that she had “chronic diarrhea” and should use loperamide as necessary. When used routinely, loperamide improved diarrhea symptoms; however, she still had significant problems with abdominal bloating, distension and frequent bouts (>1–2 episodes per week) of lower abdominal pain, cramps and severe urgency of stool. The patient, a biology teacher, has done some research and brought in a list of questions to be answered. These include: what is my diagnosis? How is the diagnosis made? Will knowing my diagnosis change my therapy? Are other tests required to make the diagnosis? What treatment options are available?

## 2. Introduction

The diagnosis of irritable bowel syndrome can be difficult for a number of reasons: one, symptoms may change over time, and these fluctuations may make the provider feel as if the disorder is more complicated than it truly is; two, symptoms of IBS may mimic other disorders (e.g., lactose or fructose intolerance) and thus may fail to respond to empiric treatment; three, providers may not be aware of current guidelines or definitions on how to properly make the diagnosis of IBS; four, a precise biomarker for IBS does not exist—patients may have persistent or recurrent symptoms but providers cannot order a test to confidently diagnose the condition; and lastly, patients may want testing to identify the cause of their symptoms, although routine tests generally result as normal, which is frustrating to the patient, since symptoms persist.

For these and other reasons, a single test with perfect sensitivity and specificity to aid in the diagnosis of IBS would be ideal. This would not just simplify the diagnosis of IBS but would enable clinicians to initiate treatment more promptly, reducing the impact of IBS to patients. Unfortunately, however, a gold standard for the diagnosis of IBS does not yet exist. Thus, clinicians and researchers have relied on a number of different criteria that have been developed over the years (e.g., Manning, Kruis, Rome), although none have proved perfect. In the section that follows, the evolution of diagnostic criteria for IBS will be reviewed.

The Manning criteria were truly the first global IBS diagnostic criteria to be introduced and have been the most extensively studied. The Manning criteria were proposed in 1978 based on symptoms thought to occur more frequently in IBS patients compared to those with organic disease [1]. In contrast to large population questionnaire studies now performed routinely, the sample size of the questionnaire study used by Manning and colleagues was quite small—only 32 patients with IBS and 33 patients with an organic disorder. The four main symptoms included looser stools at the onset of pain, increased frequency of bowel movements after the onset of pain, relief of abdominal pain after a bowel movement, and abdominal distension. Two additional symptoms were found to be of increased prevalence in patients with IBS (sensation of incomplete evacuation and fecal mucus). When 2 of 4 main symptoms were used, a sensitivity of 91% and specificity of 70% was established; when 2 of 6 symptoms were used, the sensitivity ranged from 84 to 94% and the specificity was 55%; finally when ≥3 of 6 symptoms were used, the sensitivity ranged from 63 to 90% and the specificity ranged from 70 to 93% [2]. The Manning criteria have fallen out of favor, in large part due to the fact that they do not differentiate IBS with constipation (IBS-C) from IBS with diarrhea (IBS-D), an important consideration for both drug development and for patient care.

In 1984, Kruis and colleagues reported on a similar set of symptoms used to define IBS: abdominal pain; bloating; and altered bowel function [3]. In contrast to the Manning criteria, the Kruis criteria placed a greater emphasis on symptom duration, and in fact suggested a two-year time duration. More importantly, the Kruis criteria highlighted the need to consider warning signs (“red flags”) and also to exclude organic disease with a combination of a normal physical examination and basic laboratory studies (CBC and ESR). Ultimately, however, these criteria were found to be too cumbersome to use in clinical practice and fell out of favor.

In 1988, a group of international experts met in Rome to discuss functional gastrointestinal disorders (FGIDs). An overarching goal was to classify the FGIDs using a symptom-based classification scheme, highlighting the fact that patients report symptoms despite a lack of chemical, radiological or physiological abnormalities. This culminated in the publication of the Rome criteria in 1992 (later known as Rome I), which increased the medical community’s awareness of FGIDs. Abdominal bloating, a cardinal symptom of many IBS patients, was not distinguished from abdominal pain. The criteria for IBS were easily incorporated into research studies but proved unwieldy for clinical practice. The diagnostic accuracy of the Rome I criteria was evaluated in a study of 339 IBS patients with a reported sensitivity of 85% and a specificity of 71% [4].

Several years later, the Rome committee met again to revise the initial Rome I criteria, based on feedback from clinicians, investigators, regulatory agencies and from new information gathered from the scientific literature. The revised Rome II criteria were published in 1999 [5]. Similar to Rome I, the Rome II required that symptoms be present for at least 12 weeks out of the preceding 12 months, although the time did not need to be consecutive. The term “discomfort” was added to the definition, and a new criterion was added, noting that two of the three abdominal pain-related criteria had to be required for the diagnosis of IBS to ensure that altered bowel habits were present. Patients were not categorized into specific subtypes based on bowel habits at that time.

The Rome III criteria were introduced in 2006 with the most significant change being the classification of IBS by subtypes. Subtypes were based on stool consistency rather than stool frequency, and included IBS-C (constipation), IBS-D (diarrhea), IBS-M (mixed) and IBS-U (unsubtyped). Another significant change was that the symptom of bloating as a primary symptom was eliminated from the definition [6]. This change was based on the view that bloating as a symptom is so widespread that it is neither sensitive nor specific for IBS alone. A validation study by Ford and colleagues of patients with IBS symptoms who underwent colonoscopy reported a sensitivity of the Rome III criteria as 68.8% and specificity of 79.5% [7].

Since the release of the Rome III criteria in 2006, research in the field of IBS has surged. Creative investigative work in both the basic sciences and clinical sciences identified new etiologies of IBS and provided a better understanding of the complex pathophysiology that underlies the generation of IBS symptoms [8]. A variety of new medications were introduced to the market and these focused on specific IBS subtypes, based, in part, on a better understanding of the underlying pathophysiology. These advances in knowledge, along with a desire to make the Rome criteria more clinically useful, resulted in several key changes to the Rome criteria when the fourth iteration was released in 2016 [9]. The definition and rationale for the changes are outlined below.

**Rome IV defined** irritable bowel syndrome (IBS) as a functional bowel disorder in which recurrent abdominal pain is associated with defecation or a change in bowel habits. Disordered bowel habits are typically present (i.e., constipation, diarrhea or a mix of constipation and diarrhea), as are symptoms of abdominal bloating/distension. Symptom onset should occur at least 6 months prior to diagnosis and symptoms should be present during the last 3 months (Table 1).

The Rome IV criteria (Table 1) differ from the Rome III criteria (Table 2) in several distinct ways. One, the term “discomfort” was removed from the current definition and diagnostic criteria, because some languages do not have a word for discomfort or it has different meanings in different languages. Additionally, based on a study of IBS patients who reported wide variations in their understanding of these terms, it is unclear whether the distinction between pain and discomfort is qualitative or quantitative [10]. Two, the frequency of abdominal pain was increased from 3 days per month to one day per week on average. Although this change seems small, it was based on a large population study with the goal of increasing the sensitivity and specificity of the criteria [11]. Three, bloating and distention are now recognized as common symptoms. This highlights the prevalence of these symptoms in patients with IBS and other FGIDs (i.e., chronic constipation, functional dyspepsia) and reinforces the earlier findings of Kruis and colleagues [3]. Four, the prior criteria included a somewhat ambiguous phrase regarding the presence of disordered defecation. This has now been clarified with the phrase “…disordered bowel habits are typically present (constipation, diarrhea or a mix of constipation and diarrhea)”. Lastly, it is now explicitly stated that IBS subtypes are based on predominant bowel habits on the days with abnormal bowel movements. The Rome committee, using data from a large population study (Rome Normative GI Symptom Survey; unpublished), determined that analysis of days without a bowel movement did not increase the specificity of bowel subtyping, while analyzing only days with abnormal bowel movements increased specificity.

## 3. IBS Subtypes

Classifying patients with IBS into specific subtypes based on predominant bowel habits is useful as it helps focus treatment on the predominant, and often, the most bothersome symptom. IBS is classified into four subtypes: IBS with predominant constipation (IBS-C), IBS with predominant diarrhea (IBS-D), with mixed bowel habits (IBS-M) or IBS, unsubtyped. One important change from Rome III, as noted above, is that the IBS subtype is explicitly based on the patient’s reported predominant bowel habit on days with abnormal bowel movements, and not on an average of all days, which might include days with normal bowel habits. Abnormal bowel movements are classified using the Bristol stool form scale, which is described below. For clinical trials, or when appropriate in clinical settings, subjects should complete a 14-day bowel diary to most accurately categorize IBS subtypes. Bristol stool types 1 and 2 or types 6 and 7 are considered abnormal [12].

## 4. Bristol Stool Form Scale

The Bristol stool form scale (BSFS) was developed in the 1990s in the Bristol Royal Infirmary in England [12]. The authors described seven types of stool, which are noted below:

- Type 1: Separate hard lumps, like nuts (hard to pass)
- Type 2: Sausage-shaped, but lumpy
- Type 3: Like a sausage but with cracks on its surface
- Type 4: Like a sausage or snake, smooth and soft
- Type 5: Soft blobs with clear cut edges (passed easily)
- Type 6: Fluffy pieces with ragged edges, a mushy stool
- Type 7: Watery, no solid pieces, entirely liquid

The authors classified stool types 1 and 2 as being associated with constipation, while stool types 6 and 7 were associated with diarrhea (and stool type 5 to some degree). Stool types 3 and 4 were considered normal stools. The BSFS is a convenient way for patients to describe their bowel habits, and is routinely used in clinical trials. In addition, at the two extremes (Bristol stool types 1 and 2 or types 6 and 7), the stool form serves as a rough surrogate marker of colon transit [12]. Patients with IBS-C have >25% of their bowel movements associated with BSFS 1 or 2, while those with IBS-D have >25% of their bowel movements associated with BSFS 6 or 7. Those with the mixed subtype of alternating constipation and diarrhea (IBS-M) have >25% of their bowel movements associated with BSFS 1 or 2 and >25% of their bowel movements associated with BSFS 6 or 7.

## 5. Making the Diagnosis of IBS

The illustrated case above exemplifies a typical patient with IBS. It provides us with an opportunity to apply the current Rome IV criteria in making a diagnosis of IBS. The patient describes symptoms lasting 5 years, having started after an episode of a suspected infectious food borne gastrointestinal illness. She reports current symptoms of lower abdominal pain with loose watery stools along with a sense of urgency, tenesmus, bloating and abdominal distention. She underwent stool and serum testing which was unremarkable, and reasonable trials of lactose, fructose and gluten avoidance did not help. Due to persistent symptoms, she underwent a colonoscopy with normal biopsies and was initiated on treatment to address her diarrhea; however, she was not provided with a diagnosis of IBS, and continues to have multiple questions and concerns regarding her ongoing symptoms.

Obtaining a detailed history with a few additional questions is warranted to confirm the suspected diagnosis of IBS. It is important to start by ruling out any warning signs. These include: age over 50 without prior colon cancer screening; the presence of overt GI bleeding; nocturnal passage of stools; unintentional weight loss; a family history of inflammatory bowel disease or colorectal cancer; recent changes in bowel habits; and the presence of a palpable abdominal mass or lymphadenopathy. If these warning signs are absent, further history should be obtained to quantify the frequency of symptoms and determine whether the patient meets the Rome IV diagnostic criteria. More specifically, the patient should be asked if her pain is present at least one day a week on average for the last 3 months. In our case, the patient has reported a duration of 5 years, which meets the requirement of having an onset greater than 6 months prior to diagnosis. The rationale behind the latter two questions is to ensure that the symptoms are recent, and that there is no organic disease manifesting itself over at least 6 months. The final component to applying the criteria involves associating the abdominal pain to bowel habits. A careful history should be obtained to confirm whether abdominal pain is related to defecation, a change in stool frequency, or a change in the appearance of stool. In order to clarify the latter characteristic, the Bristol stool chart should be employed as previously described. A benign physical examination further supports the diagnosis of IBS, although the importance of a physical examination cannot be underestimated as this does reassure the patient [8,9].

As illustrated in this case, unfortunately the yield of confirmatory testing to rule out an alternate diagnosis is low. This highlights the rationale outlined in position statements, original articles, and review articles that extensive testing in patients with symptoms of IBS who meet Rome criteria is unlikely to uncover a new diagnosis [8,9]. It is important, however, to obtain a complete blood count to ensure the absence of an iron deficiency anemia, and a CRP can be requested to lower the suspicion for inflammatory bowel disease. Alternatively, a fecal calprotectin can be considered, especially in IBS patients with diarrhea or with diarrhea and constipation, since it can help differentiate IBS from IBD with good accuracy and may prevent the indiscriminate use of colonoscopy. Celiac testing should be obtained, ideally in the setting of adequate gluten consumption, since IBS may mimic this disorder. At this point in the evaluation, if patients meet the diagnostic criteria for IBS, further testing should be discouraged and education and reassurance provided. Based on a prospective case-control study including 466 patients, colonoscopy did not change the diagnosis of IBS in 98.1% of patients [13]. Separate trials of avoiding lactose, fructose and/or gluten as diagnostic maneuvers can be considered in case symptoms are related to an intolerance to these foods. These trials can be carried out under the supervision of a dietician to avoid over restriction, ensure nutritional adequacy and implement strategies that integrate with the patients’ habitual diet. Alternatively, a low-FODMAP diet (an acronym derived from Fermentable, Oligo-, Di-, Mono-saccharides And Polyols) can be instituted to eliminate many possible culprit foods all at once [14,15].

## 6. Case Study: Management

At the initial consultation visit, the first step was to carefully listen to the patient’s story. One of the most important tools to ensure a satisfactory patient visit is to allow time to let the patient tell their story. This helps set a strong foundation in building a strong physician–patient relationship. After the patient provides her history, it may be useful to briefly recapitulate the history, as this lets the patient know that attention is being paid and also offers a chance to correct any misinterpretation of the patient’s history. As a part of this process, it is important to review all prior diagnostic studies and treatments. This is time well-spent, as it will prevent unnecessary repeat testing or therapeutic trials. It is also important to ask patients whether they have any fears or concerns about their symptoms, as many patients with IBS symptoms are quite concerned that their symptoms represent a hidden malignancy or IBD [16]. A brief physical examination should be performed; this too reassures the patient that complaints are being taken seriously. In this patient, who does not have predominant symptoms of constipation, a rectal examination is not required, especially since she recently had a colonoscopy that was normal. However, in patients with constipation symptoms, and certainly in those without recent colonoscopic evaluation, a careful rectal examination should be performed. This is useful to help diagnose patients with pelvic floor dyssynergia.

At this point, the patient should be confidently told that she has IBS. She clearly meets Rome IV criteria. Symptoms have been present for greater than 6 months and have been active for the last 3 months. She suffers from abdominal pain more than 1 day per week on average, and pain is temporally related to disordered defecation and to a change in stool appearance and frequency. There are no warning signs on history or examination. In addition, prior diagnostic testing, including a colonoscopy with biopsies, and serologic tests to rule out celiac disease, have all been normal. She should also be told about the possible etiology to her developing IBS, which appears to be post infectious in nature. It is important that patients walk away from their visit with a confident diagnosis, as two other key components of a successful patient visit include educating and reassuring the patient about their condition. However, education and reassurance cannot occur if a diagnosis is not made. Furthermore, language that communicates diagnostic certainty is essential, since it conveys confidence in the diagnosis and allows acceptance by the patient, thus preventing further unwarranted testing [17]. Just as essential, making the diagnosis of IBS with appropriate subtyping, based on an understanding of the patient’s predominant symptoms, will help guide appropriate therapy.

For this patient, she is told that the diagnosis of IBS is based on a constellation of symptoms, an absence of warning signs, a normal physical examination, and the results of limited diagnostic tests. The Rome IV criteria can be explained in terms comfortable to the patient. For this patient, who is quite savvy, knowing that she meets specific criteria for IBS should be reassuring. In addition, it will provide her with the appropriate framework to do on-line research on her own. At this point, the patient should be confidently told that no further testing is required. Extensive testing is unlikely to uncover an alternative diagnosis and will not reassure the patient. In fact, subjecting each and every patient with IBS symptoms to a battery of expensive, and sometimes dangerous, tests only undermines their confidence in the ordering provider. The fourth key component of a successful patient visit involves working together to improve symptoms. As mentioned, the key symptom (or symptoms) should be identified and treatment initiated. In this case, the patient appears to have IBS with predominant diarrhea and therapy can be tailored accordingly. For instance, a low-FODMAP diet could be initiated with the help of a dietician if she wanted to start with dietary interventions. Alternatively, a gut-directed antibiotic such as rifaximin would be a very reasonable choice as well [8,9,18]. If symptoms of abdominal pain persist, a low dose tricyclic antidepressant should be initiated. This should improve visceral pain and may slow colonic transit to some degree [19]. If symptoms persist, subsequent medication trials could include alosetron, eluxadoline or a bile acid sequestrant [8,9]. Interventions such as gut directed hypnotherapy and cognitive behavioral therapy can also be considered in select patients [20]. The patient should be asked to call the office for a quick follow-up approximately 4 weeks after initiating therapy. It is also important to schedule a follow-up visit in the office to answer new questions, continue to reassure and educate the patient, and fine-tune dietary or medical therapy.

## 7. Conclusions

Establishing the diagnosis of IBS can be challenging since there is no confirmatory test. The development of criteria since 1978, with its most recent iteration in 2016, have sought to clarify and aid practitioners in making the diagnosis. A careful history is key to a cost-effective diagnosis of IBS and patients meeting the Rome IV diagnostic criteria, with a normal physical exam and the absence of any warning signs, should have only limited testing as outlined above. They should be discouraged from repeated testing and be provided with reassurance and education instead. The Bristol stool chart should also be used to objectively describe bowel habits and classify patients into the correct subtype in order to direct treatment according to the predominant symptom.

## Figures and Tables

**Table 1:** IBS Diagnostic Criteria *.

Recurrent abdominal pain on average at least 1 day/week in the last 3 months, associated with two or more of the following criteria:
1. Related to defecation
2. Associated with a change in the frequency of stool
3. Associated with a change in the form (appearance) of stool

(These criteria should be fulfilled for the last 3 months with symptom onset at least 6 months prior to diagnosis.) * Modified from Rome IV [9].

**Table 2:** Previously used Rome III Diagnostic Criteria for Irritable Bowel Syndrome [6].

Recurrent abdominal pain or discomfort (defined as an uncomfortable sensation not described as pain) for at least 3 days/month in the last 3 months, associated with two or more of the following:
1. Improvement with defecation
2. Onset associated with a change in the frequency of stool
3. Onset associated with a change in the form (appearance) of stool

These criteria should be fulfilled for the last 3 months with symptom onset at least 6 months prior to diagnosis.
